# Long Sleep Duration and Social Jetlag Are Associated Inversely with a Healthy Dietary Pattern in Adults: Results from the UK National Diet and Nutrition Survey Rolling Programme Y1–4

**DOI:** 10.3390/nu10091131

**Published:** 2018-08-21

**Authors:** Suzana Almoosawi, Luigi Palla, Ian Walshe, Snieguole Vingeliene, Jason G. Ellis

**Affiliations:** 1 Brain Performance & Nutrition Research Centre, Faculty of Health and Life Sciences, Northumbria University, NE1 8ST Newcastle-upon-Tyne, Tyne and Wear, UK; 2 Faculty of Epidemiology and Population Health, London School of Hygiene & Tropical Medicine, WC1E 7HT London, UK; Luigi.Palla@lshtm.ac.uk; 3 Department of Sport, Exercise and Rehabilitation, Faculty of Health and Life Sciences, Northumbria University, NE1 8ST Newcastle-upon-Tyne, Tyne and Wear, UK; ian2.walshe@northumbria.ac.uk; 4 Clinical Epidemiology and Biostatistics, School of Medical Sciences, Örebro University, 702 81 Örebro, Sweden; snieguole.vingeliene@oru.se; 5 Northumbria Sleep Research Laboratory, Faculty of Health and Life Sciences, Northumbria University, NE1 8ST Newcastle-upon-Tyne, Tyne and Wear, UK; jason.ellis@northumbria.ac.uk

**Keywords:** sleep, social jetlag, diet food and nutrition, nutrition surveys, cross-sectional, epidemiology, adults, public health

## Abstract

Limited observational studies have described the relationship between sleep duration and overall diet. The present study investigated the association between sleep duration on weekdays or social jetlag and empirically derived dietary patterns in a nationally representative sample of UK adults, aged 19–64 years old, participating in the 2008–2012 UK National Diet and Nutrition Survey Rolling Programme. Survey members completed between three to four days of dietary records. Sleep duration on weekdays was categorized into tertiles to reflect short, normal, and long sleep duration. Social jetlag was calculated as the difference between sleep duration on weekends and weekdays. The association between sleep duration/social jetlag and dietary patterns, derived by principal components analysis, was assessed by regressing diet on sleep, whilst accounting for the complex survey design and adjusting for relevant confounders. Survey members in the highest tertile of sleep duration had on average a 0.45 (95% Confidence Interval (CI) −0.78, −0.12) lower healthy dietary pattern score, compared to middle tertile (*p* = 0.007). There was an inverted u-shaped association between social jetlag and the healthy dietary pattern, such that when sleep on weekends exceeded weekday sleep by 1 h 45 min, scores for indicating a healthy dietary pattern declined (*p* = 0.005). In conclusion, long sleep duration on weekdays and an increased social jetlag are associated with a lower healthy dietary pattern score. Further research is required to address factors influencing dietary patterns in long sleepers.

## 1. Introduction

Sleep has been a relatively neglected component in nutritional epidemiology, with limited nutritional surveys and cohorts collecting data on sleep timing, duration, or quality [1,2]. In recent years, however, sleep has emerged as a potential critical modulator of metabolic pathways involved in glucose homeostasis [3], energy metabolism, and appetite regulation [4,5]; as well as a potential important modifiable factor influencing eating behavior [5,6], nutritional status and diet quality [7]. Against this, multiple cross-sectional studies emerged describing associations between short or long sleep duration and poorer nutritional status and diet quality, though the direction of the association is yet to be established due to limited longitudinal evidence [2,7]. In a cross-sectional analysis of the UK National Diet and Nutrition Survey Rolling Programme (NDNS RP), British adults with a sleep duration ranging from seven to eight hours reported higher intakes of vitamin C, fiber and iron, and higher levels of serum total carotenoids, selenium, and urinary nitrogen, compared to short (≤6 h/night) or long sleepers (≥9 h/night) [2]. Similarly, normal sleep duration has been associated with higher fruit and vegetable intake in one analysis of the NDNS RP [8], but not in another [9]. The latter inconsistency may arise from differences in the methodologies used to analyze dietary data, wherein most of the focus has been on single foods or nutrients.

In the past, investigating associations between single foods or nutrients has provided invaluable insight into potential molecular pathways, by which food may influence health or vice versa [10]. Yet, it is recognized that intake of one food group or nutrient may correlate with intake of other food groups or nutrients [10]. Thus, analyses focusing on single foods or nutrients may potentially mask associations with more relevant foods or nutrients [11]. Indeed, foods and nutrients are often consumed in combination and form part of complex dietary patterns, for which they act as markers [11]. Exploring the association between sleep and dietary patterns is essential, since dietary strategies based on dietary patterns are more easily deployable in a public health framework and have been shown to be more effective, compared to strategies focusing on specific foods or nutrients [10,12]. To date, few studies have described the relationship between sleep duration and overall dietary patterns [13]. Moreover, to our knowledge, no study has addressed how variations between sleep duration on weekends and weekdays, referred herein as social jetlag, may relate to overall diet. The latter is important considering that social jetlag is a common feature of the modern society [14] wherein, under the constraints of social and working schedules, individuals sleep for shorter durations during weekdays, compensated by longer sleep duration on weekends [15,16,17]. Given the known adverse effects of social jetlag on human health [14], accounting for sleep duration on weekends and investigating the association between social jetlag and diet may be important in informing future policies, in terms of both sleep and diet.

The present study investigated the association of sleep duration and social jetlag with empirically derived dietary patterns in a nationally representative sample of UK adults.

## 2. Methods

### 2.1. Study Population

Excluding the variable for mental illness, the study population consisted of a complete-case sample size of 2433 adults, aged 19–64 years old, who participated in the 2008–2012 NDNS RP, and who completed three or four days of dietary assessment [18]. The NDNS RP is a cross-sectional rolling survey, which collects information on all food and drinks consumed from a representative sample of the British population including, at each round, approximately 1000 randomly sampled individuals, living in private households across the four regions in the UK, i.e., England, Scotland, Wales, and Northern Ireland [18].

Individuals were selected from a random sample of 21,573 addresses from 799 postcode sectors, obtained between April 2008 and March 2011, from the Royal Mail’s Postcode Address File. Within each selected address, one household was randomly sampled. The overall response rate for individuals completing three or four days of dietary records was 56% in Year 1, 57% in Year 2, 53% in Year 3, and 55% in Year 4, respectively. Details of the survey methodology have been published previously [18]. Ethical approval for the NDNS RP was obtained from Oxfordshire Research Ethics Committee [18], and data obtained from the UK data archive (www.ukdataservice.ac.uk, accession number 123803).

### 2.2. Dietary Assessment

Interviewers visited participants in their home, wherein they provided an estimated food diary to be completed over four consecutive days by survey members [18]. Survey members were provided with written instructions and asked to record everything they ate and drank over the four days, both at home and outside. To ensure compliance and completeness of recording, follow-up checks were scheduled by the interviewer on the second or third day of recording, either in person or by telephone [18]. Home visits were carried out continually throughout each year, from February 2008 to August 2012, to ensure that seasonal variations in dietary intake were captured [18]. Diary entries were coded and analyzed by trained dietary coders, using an in-house dietary assessment system DINO (Data In, Nutrients Out) [18]. This system is based on food composition data from the Department of Health’s NDNS Nutrient Databank, which contains over 7000 regularly updated food codes [18].

### 2.3. Sleep

Data on sleep were collected by asking each individual survey member: “Over the last seven days, that is since last (seven days), how long did you usually sleep for on week nights. That is Sunday to Thursday nights?”, “And over the last seven days, how long did you usually sleep for on weekend nights. That is Friday and Saturday nights?” [19]. If the respondent worked on night shifts during the last one week, the average time slept during the day was recorded. If the pattern of time spent in sleep varied widely, interviewers coded the response as ‘don’t know’. In Year 1–2, duration of sleep was recorded into a single variable, reflecting time spent asleep in hours and minutes [19]. In Years 3–4, sleep duration was recorded into two separate variables, one to reflect the hours spent asleep, and the second to reflect minutes. To enable data analyses from all years, a new sleep variable was derived to reflect time spent in sleep in hours and decimal fraction of hours. Given that the association between sleep and health [20] or diet may be non-linear [21], the derived sleep duration variable was split into tertiles, to generate three sleep categories based on the sleep duration distribution of the survey sample.

Social jetlag was calculated as the difference in sleep duration between weekends and weekdays, in hours and decimal fraction of hours.

### 2.4. Additional Measures and Covariates

Height and weight were measured using a portable stadiometer and weighing scales. Body Mass Index (BMI) was calculated as weight in kilograms divided by height in meters squared. A Computer Assisted Personal Interview (CAPI) was also conducted during the initial visit by trained interviewers, to obtain information on respondents health status including limiting long-term illnesses; smoking habits (current smoker, ex-regular smoker, never smoker); socio-economic characteristics; and ethnicity (white vs. non-white). Socio-economic status was defined based on the National Statistics Socio-economic Classification (NS-SEC) as: (1) Managerial and professional occupations; (2) Intermediate occupations; (3) Small employers and own account workers; (4) Lower supervisory and technical occupations; (5) Semi-routine and routine occupations; and (6) Never worked and long-term unemployed. A variable for mental illness was calculated and coded as ‘Yes’ or ‘No’, based on self-reported presence or absence of limiting long-term illness. Briefly, survey members were asked ‘Do you have any long-standing illness, disability, or infirmity? By longstanding I mean anything that has troubled you over a period of time, or that is likely to affect you over a period of time?’ If survey members answered ‘Yes’, then an illness was selected from a pre-defined drop-down list. Mental illness included depression, anxiety, or nervousness.

### 2.5. Statistical Methods

#### 2.5.1. Principal Component Analysis

Within the sample included in the analyses, seven survey members reported consuming foods within the ‘Commercial Toddlers Foods and Drinks’ food group. These foods were inspected and recoded into more relevant main food groups, which were ‘Biscuits’ or ‘Crisps and savory snacks’, so as not to exclude any survey members′ data. A total of 60 main food groups were included in the Principal Component Analysis (PCA), to derive dietary patterns. These food groups reflected the main food group classification used in the NDNS RS. Details of the food group descriptions including definitions of ‘other’ have been published previously, in Appendix R of the full report [18]. Data were inputted as mean daily intake in grams (g/day) for each individual. PCA produced linear combinations of food groups that account for the highest possible variance in the data set and are mutually independent. They are deemed to represent discrete dietary patterns specific to the population analyzed. Varimax rotation was applied to derive the most optimal uncorrelated food patterns, and to enhance the representation of food groups in each dietary pattern. Scree plots were used to plot eigenvalues, to visualize the amount of variance captured by the dietary patterns. Based on this, only the initial three components were retained, which had an Eigenvalue above 1.5, and which together accounted for 11% of the variance in food intake. The eigenvalue threshold at 1.5 was also consistent with the approach followed in a previous study using NDNS data [22].

A dietary pattern score was calculated for each survey member, for each of the derived dietary patterns. These scores were calculated by multiplying the component loadings by the corresponding standardized weight for each food and summing the values across the food groups. A higher score indicated closer adherence to the corresponding dietary pattern. Dietary patterns were named based on food groups, with component loadings above the 0.2 or below −0.2 threshold. Such food groups were deemed to have a stronger influence on the respective dietary pattern and were most informative in describing dietary patterns.

#### 2.5.2. Multiple Regression

Differences in sociodemographic characteristics and dietary intake between survey members across sleep duration tertiles on weekdays, were assessed using one-way analysis of variance (ANOVA) for continuous variables, or *χ*^2^ tests for categorical variables. These analyses were adapted for complex survey design using the variables for clusters, strata, and individual weights. The centered method was used to deal with singleton units, in the initial stage of the analysis.

Associations between sleep duration or social jetlag as exposure variables, and dietary patterns as outcomes, were investigated using multiple regression models accounting for complex survey design. Data were weighted to correct for unequal sample selection, non-response for household and individual interview, and non-response to individual visit [23]. This weighing factor adjusted for differences in socio-demographic variables, such as age, sex, ethnicity, and region, between participants and non-participants to the individual visit to ensure the survey sample was representative of the UK population [23]. Analyses were conducted both using the centered method for complex survey design and by re-assigning singleton units to the nearest Primary Sampling Unit. Only results for the models with the re-assigned singleton units are presented. The crude model included only the variables for sleep duration on weekdays and weekends. Model 1 adjusted for sex, ethnicity, NS-SEC, age, smoking status, and mean daily energy intake. Model 2 additionally adjusted for BMI, while Model 3 further included mental illness as a covariate. These covariates were selected as potential confounders, based on their association with sleep and potential influence on diet. Overall, 108 (4%), 110 (4%), 7 (0.03%), 340 (13%), 173 (6%), and 1809 (67%) survey members had missing data on sleep duration on weekdays, sleep duration on weekends, NS-SEC, BMI, and mental illness, respectively. These values were assumed to be missing at random and were consequently imputed using multiple imputations. Fifty imputed data sets were created and fitted by using the “ice” and “mim” packages in Stata MP version 13 (StataCorp LP, College Station, TX, USA) [24]. This gave an imputed sample of 2697 survey members. Sensitivity analyses were conducted on Model 2, comparing the coefficients derived from imputed data and the complete case analyses. Additional sensitivity analyses, including interactions between sleep duration on weekdays or social jetlag and BMI, age, or mental illness were conducted. The significance of interactions was assessed by Wald tests and the global predictive power of different models by *R*^2^, using the mibeta command. These interactions were subsequently dropped from the final model. Sensitivity analyses using sleep duration expressed as a quadratic continuous term, or as a categorical term defined as short sleep duration (≤6 h/night), normal sleep duration (7–8 h/night), and long sleep duration (≥9 h/night) were also conducted.

In relation to social jetlag, analyses were conducted using models with social jetlag expressed, either in tertiles or as a quadratic continuous term. Model fit was better for the model including the quadratic term. Thus, the final model included a linear and quadratic term for social jetlag, sex, ethnicity, NS-SEC, smoking, age, BMI, and total energy intake.

All statistical analyses were carried out using Stata Statistical Software version 13 (StataCorp LP, College Station, TX, USA). To reduce the effect of multiple testing on type I error, a more stringent *p*-value of ≤ 0.01 was deemed significant for all tests.

## 3. Results

### 3.1. Sleep Duration and Social Jetlag

Characteristics of the complete-case study sample, are provided in Table 1, according to tertiles of sleep duration on weekdays. Overall, survey members within the lowest tertile of sleep duration (short sleep) during weekdays reported a mean 6.3 h (standard deviation (SD) ± 0.9) of sleep, compared to 7.8 h (SD ± 0.2) and 9.2 h (SD ± 0.7) of sleep reported by survey members in the middle (normal sleep) and highest tertile (long sleep), respectively (*p* < 0.001). Compared to individuals within the lower and middle tertile, survey members within the highest tertile were more likely to be of a younger age (*p* < 0.001) and have lower BMI (tertile 3: 26.6 kg/m^2^ (SD ± 5.3) vs. tertile 1: 28.2 kg/m^2^ (SD ± 5.5) *p* < 0.001). There was a tendency for women to be in the highest tertile of sleep (70% of women vs. 31% of men, *p* = 0.017). Sleep duration on weekends was correlated with sleep duration on weekdays, such that a higher proportion of short, normal, and long sleepers on weekdays were also short, normal, or long sleepers on weekends (*p* < 0.001). Survey members in the lowest tertile of sleep duration on weekdays reported sleeping on average 36 min (SD ± 1 h 12 min) more on weekends, compared to those in the highest tertile of sleep who reported sleeping 12 min (SD ± 1 h 12 min) less during weekends (*p* < 0.001).

### 3.2. Diet and Dietary Patterns

Univariate analyses (*χ*^2^), showed statistically significant association between the sleep duration tertiles and the proportion of consumers vs. non-consumers of ‘beer, lager, cider, perry’ (*p* = 0.003). There was a tendency for the proportion of consumers of ‘nuts and seeds’ (*p* = 0.019), ‘oily fish’ (*p* = 0.018), ‘brown, granary, and wheatgerm bread’ (*p* = 0.020), ‘dry weight beverages’ (*p* = 0.026), and ‘fruit’ (*p* = 0.040) to be lower in the upper tertile of sleep, while there was a tendency for a higher consumption of ‘white fish, coated or fried’, compared to the lower tertile of sleep duration (*p* = 0.027). These differences, however, did not reach statistical significance (Table 2).

**Figure 1:**
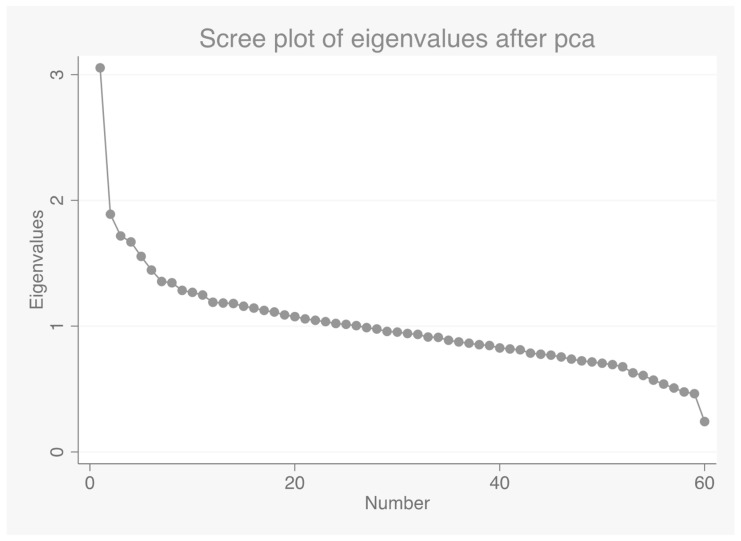
Scree plot for identification of dietary patterns by principal component analysis. Food intakes (g/day) were aggregated into 60 food groups. An eigenvalue >1.5 was selected as the cut-off for retaining the components.

**Figure 2:**
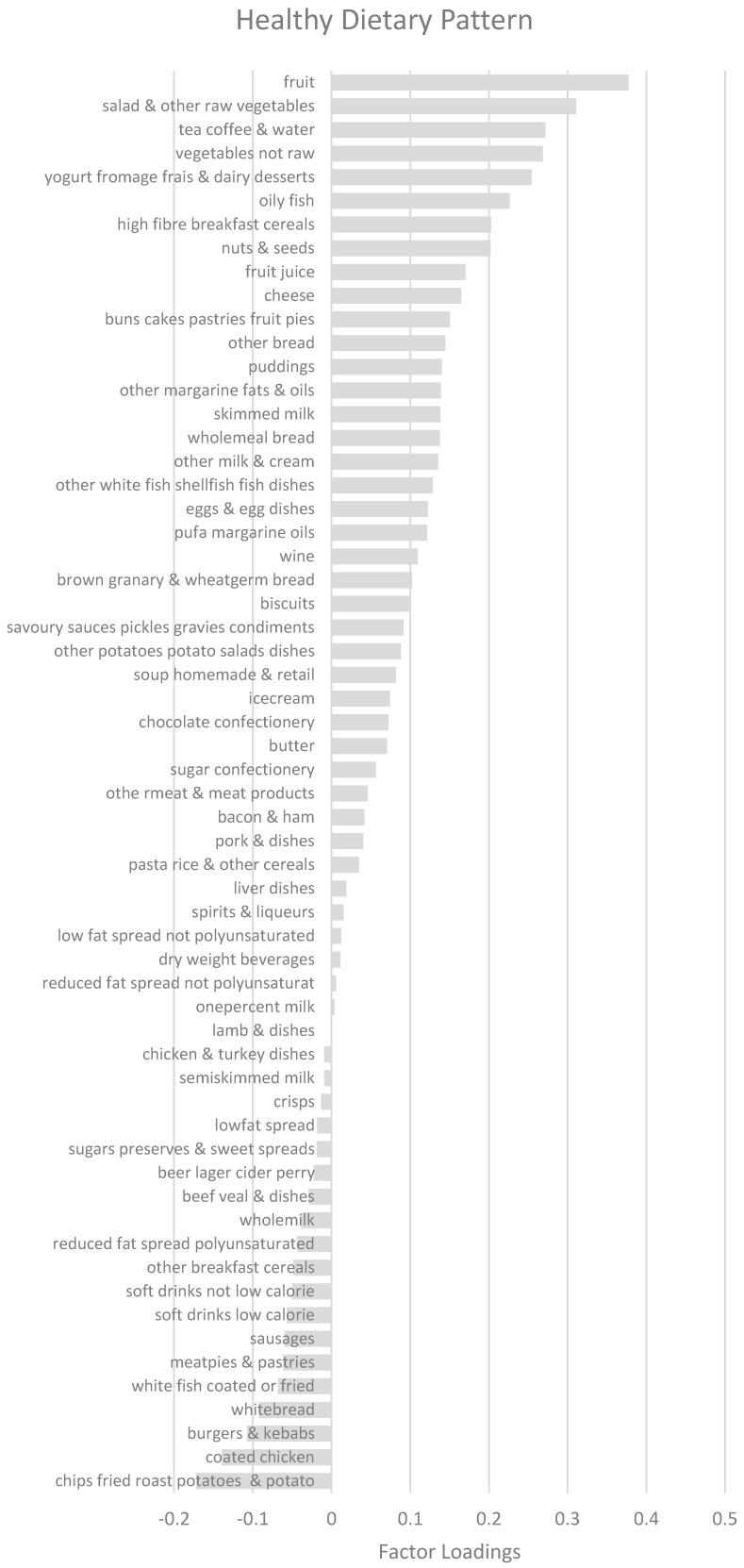

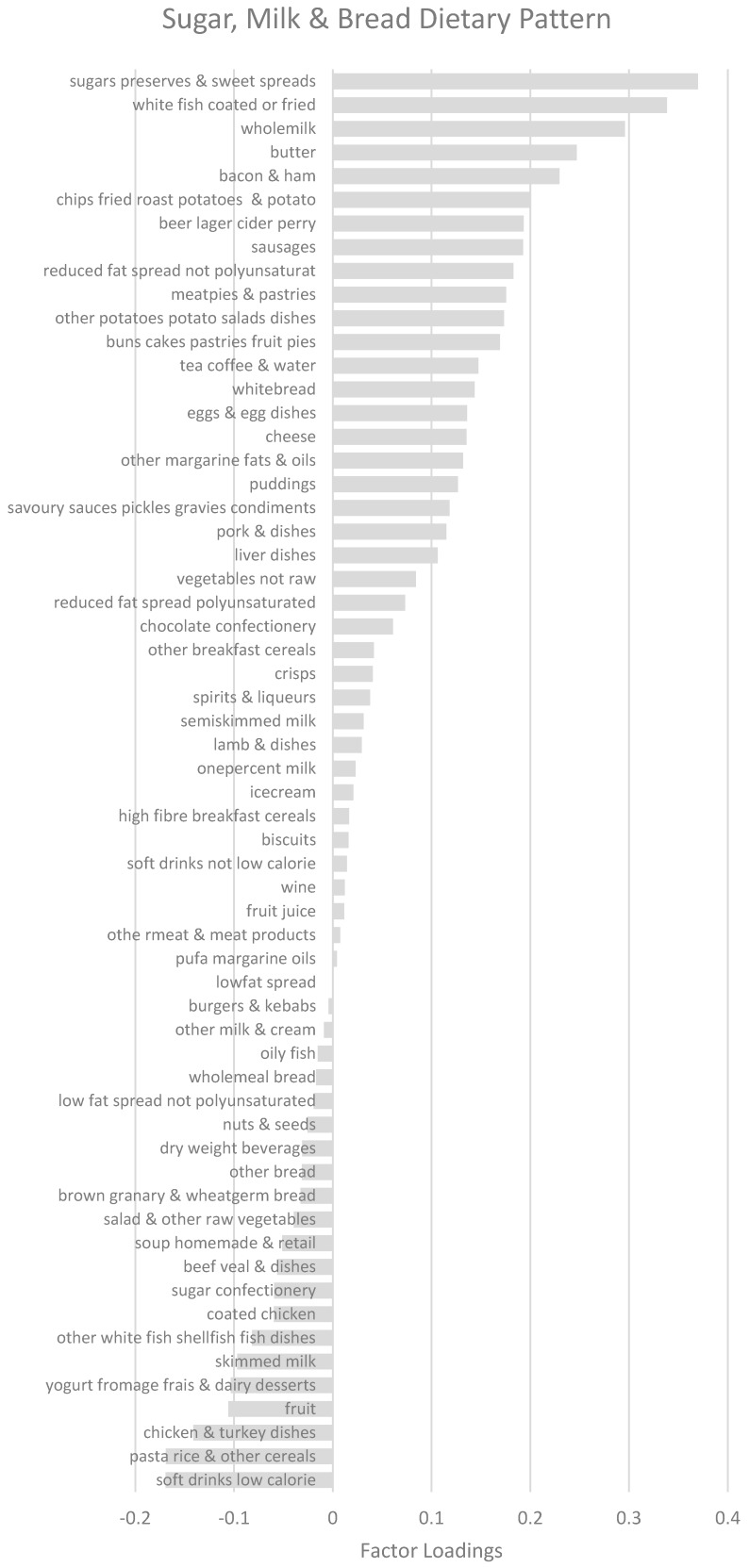

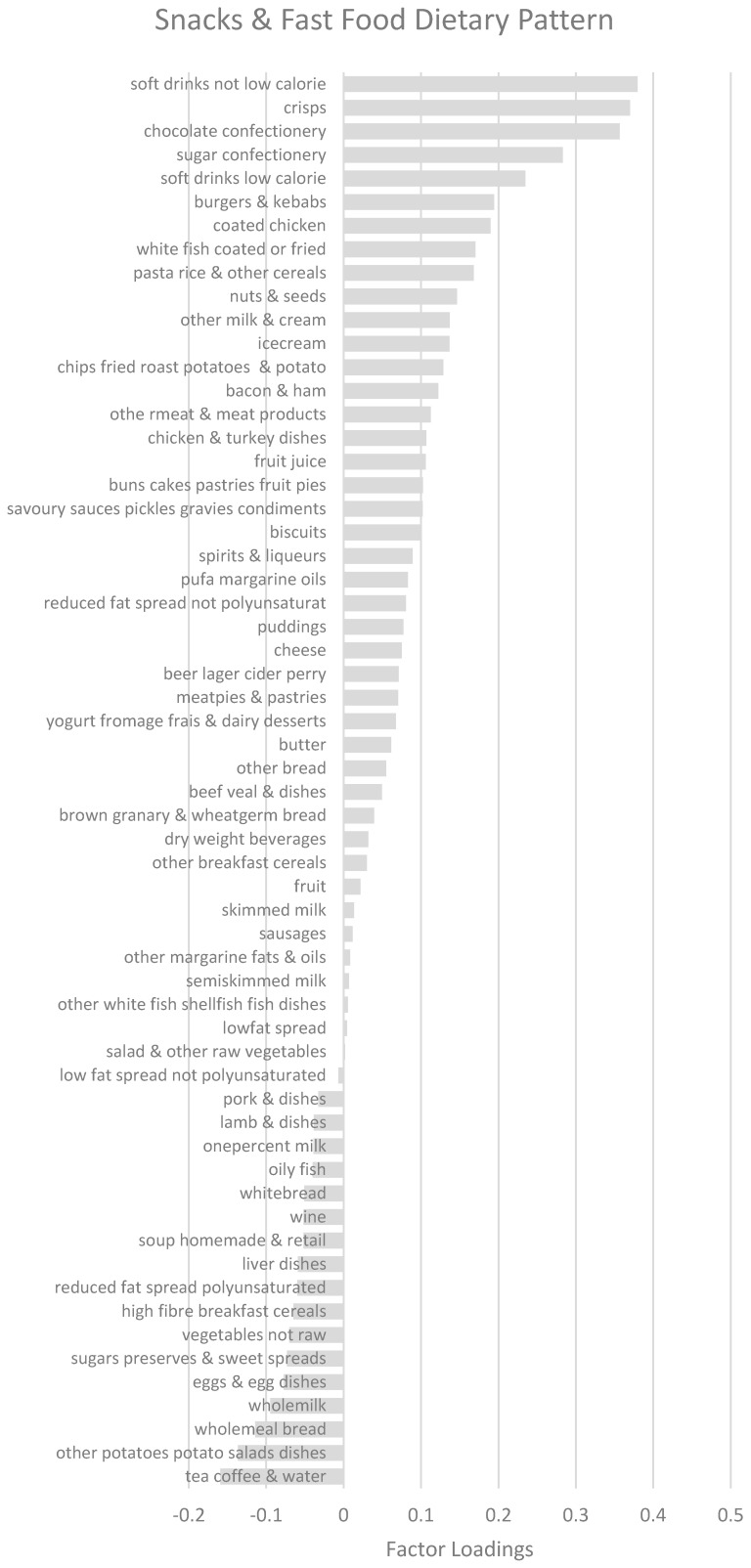
Factor loadings for dietary patterns. Abbreviations: PUFA, Polyunsaturated fatty acids.

Three dietary patterns were derived using PCA, accounting together for 11% of variance of food intake (Figure 1). The first dietary pattern accounted for the largest proportion of variation in food intake (5.0%) (Figure 2). This dietary pattern resembled a ‘healthy’ dietary pattern and had positive correlations with the food groups: ‘fruit’, ‘salad and other raw vegetables’, ‘tea, coffee, and water’, ‘vegetables not raw’, ‘yoghurt, fromage frais and dairy dessert’, ‘oily fish’, ‘high-fiber breakfast’, and ‘nuts and seeds’.

The second dietary pattern explained 3.0% of the variance of food intake and was termed ‘sugar, bread, and milk’ dietary pattern. It had positive correlations with ‘sugar, preserves, and sweet spreads’, ‘white bread’, ‘whole milk’, ‘butter’, ‘bacon and ham’ and ‘chips, fried and roasted potatoes’.

The third dietary pattern resembled a ‘snacks’ dietary pattern and explained 3.0% of the variance. It had positive correlations with ‘soft drinks not low calorie’, ‘crisps and savory snacks’, ‘chocolate confectionery’, ‘sugar confectionery’, and ‘soft drinks low calorie’.

### 3.3. Sleep and Relationship with Dietary Patterns

Results for the multiple regression models of the association between sleep duration on weekdays and the healthy dietary pattern as the outcome variable, are shown in Table 3. In the unadjusted models, a longer sleep duration was associated with a lower healthy dietary pattern score (β −0.60, 95% Confidence Interval (CI) −0.95, −0.26, *p* = 0.001). After adjustment for initial covariates in Model 1, the inverse association between a longer sleep duration and the healthy dietary pattern remained significant (β −0.48, 95% CI −0.82, −0.15, *p* = 0.005). This association was significant even after adjustment for BMI in Model 2 (β −0.45, 95% CI −0.78, −0.12, *p* = 0.007) and mental illness (β −0.44, 95% CI −0.77, −0.11, *p* = 0.009—Model 3 included in Supplementary Material). Removing sleep duration on weekends from the Model 2, resulted in a decrease in the value of the coefficient for the association between the upper tertile of sleep duration on weekdays and the healthy dietary pattern, (Data included in Supplementary Material). Compared to the model with complete cases, Model 2 with imputed data had a similar coefficient for the long sleep duration tertile, but a narrower confidence interval (Table 3). Using alternative sleep duration cut-offs did not change the value of the coefficient, (Data included in Supplementary Material). However, the global predictive power of the model as assessed using *R*^2^ was reduced.

There was a tendency for long sleep duration on weekdays to be positively associated with the sugar, bread, and milk dietary pattern (β 0.29, 95% CI 0.06, 0.51, *p* = 0.013). No significant association between sleep duration and the ‘snacks’ dietary pattern were observed (β −0.13, 95% CI −0.37, 0.11, *p* = 0.300).

**Figure 3:**
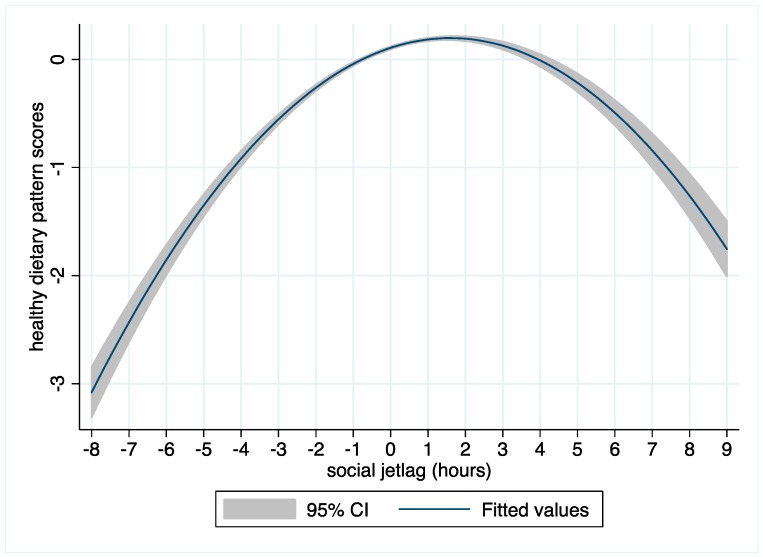
Inverse u-shaped relationship between social jetlag and the fitted values (with confidence bands), for the healthy dietary pattern scores from the multiple regression model 2.

There was a significant concave nonlinear association between social jetlag and the healthy dietary pattern (Table 4). As shown in Figure 3, the positive association between social jetlag and the healthy dietary pattern was levelled, when sleep duration on weekends exceeded sleep duration on weekdays by 1 h 45 min, reflecting the vertex of the curve. The association between social jetlag and the healthy dietary pattern reversed beyond this point, such that any further increase in sleep duration on weekends, compared to weekdays, was associated inversely with the healthy dietary pattern. Using Figure 3 and two time periods, between 2 to 3 h and 3 to 4 h as reference points, it can be observed that the degree of change in the healthy dietary pattern score was greater for the latter social jetlag increase, despite the same time lapse. This association remained significant after adjustment for potential covariates, including BMI and mental illness (β for quadratic term −0.03, 95% CI −0.04, −0.01, *p* = 0.006). No associations between social jetlag and the remaining dietary patterns were observed, (data not shown).

## 4. Discussion

### 4.1. Main Findings

The current study addressed a major gap in the research literature on sleep and nutrition, by investigating the relationship between sleep duration and overall dietary patterns in a nationally representative sample of UK adults. Addressing this research gap was important, since previous research has focused on describing the relationship between sleep duration and individual nutrients or foods [7]. Such a reductionist approach often ignores complex interconnections between the various food groups, thereby potentially undermining more relevant associations and rendering a public health campaign centered on sleep and diet challenging. In the present study, we identified an inverse relationship between long sleep duration on weekdays and a healthy dietary pattern characterized predominantly by higher intakes of ‘fruit’, ‘salad and other raw vegetables’, ‘tea, coffee, and water’, ‘vegetables not raw’, ‘oily fish’, ‘high-fiber breakfast’, and ‘nuts and seeds’. This association remained significant even after adjustment for relevant confounders including BMI, mean daily energy intake, and sleep duration on weekends. In terms of public health implications, our findings may have important value, given that a decline of 0.45 units in the healthy dietary pattern score can be translated as a mean daily increase of approximately 500 kcal in energy intake, accompanied by a 22 g increase in the intake of ‘chips, fried, roasted potatoes, and potatoes’ at the expense of a 2-portion reduction in daily fruit intake.

Our findings contrasted with a previous analysis of the NDNS RP, wherein sleep duration was not found to be related to diet [9]. This may be ascribed to the single-nutrient/food approach, adopted by the previous analysis [9]. Our study, however, complements the observations made by Dashti who found that normal sleep duration was associated with more favorable dietary behavior [25]. Our findings were also consistent with previous observations made by Mossavar-Rahmani and colleagues, who found that long sleepers had lower intake of caffeine in a sample of 16,415 Hispanic/Latino participants living in the US [26]. Similar observations have been made by Grandner, who reported lower intake of theobromine, a metabolite of caffeine, and marker of tea and coffee intake in long sleepers [27]. Contrary to Grandner and colleagues [27], however, we did not observe a u-shaped relationship between sleep duration and energy intake in the univariate analysis. Similarly, we did not observe an inverse association between short sleep duration and the healthy dietary pattern. This contrasts with previous research, which has demonstrated associations between short sleep duration and reduced fruit [28,29] and/or vegetable intake [28]. In the Shanghai Women′s Health Study, which included 68,832 Chinese women, an inverse association between short sleep duration (<6 h) and tea and fruit intake was also found [30]. In our study, the lack of findings of an association between short sleep duration and dietary patterns may be explained by differences in the cut-offs used for defining the sleep duration tertiles, as well as the lack of objective measures of sleep. In the former case, redefining the tertiles using 6 h or 9 h as the cut-off for the lower or upper tertile, respectively, did not alter the findings. Concerning measures of sleep, we were unable to differentiate between short sleep duration associated with circadian misalignment and sleep disturbance, versus short sleep that is not associated with circadian misalignment and sleep disturbance. The latter is important, considering that the combination of short sleep and circadian misalignment is associated with more adverse health outcomes [31]. Moreover, growing evidence suggests that sleep duration, sleep quality, and social jetlag may be modulated by an individual’s circadian type or so-called chronotype [32]. Accordingly, in a study of obese short sleepers, Lucassen and colleagues found that only individuals with an evening chronotype were more likely to have an unhealthy eating pattern characterized by larger food portions, less frequent meals, and greater energy intake later in the day [33]. This may imply the need for controlling for chronotype in nutrition epidemiological studies investigating the association between sleep duration and diet.

In the present study, no significant association between shorter sleep duration and the ‘snacks’ dietary pattern was observed. This was in contrast to intervention studies, which have reported increased intake of snacks [34,35], particularly carbohydrate-rich snacks and dessert, following sleep restriction [35,36]. This was also in contrast to epidemiological studies, which have described a positive association between short sleep duration and snack intake after dinner [1,37]. Future analyses of the NDNS RP should attempt to examine differences in food intake, according to time-of-day, using methods such as correspondence analysis [38]. Differences in eating occasion-based dietary patterns may likewise warrant exploration [21].

A novel finding of the current study was the inverted u-shape relationship between social jetlag, defined in the present study as the difference between sleep duration on weekends and weekdays, and the health dietary pattern. We found that beyond a 1 h 45 min positive social jetlag on weekends, scores for the healthy dietary pattern declined. These findings were interesting as they, albeit speculatively, indicated that sleep compensation on weekends was associated with an improved diet, up to a certain threshold. Although we cannot speculate on the nature of this relationship, this analysis was further supported by the sensitivity analyses we conducted, wherein the negative relationship between long sleep duration on weekdays and the health dietary pattern was attenuated, when sleep duration on weekends was not accounted for. Such findings suggest the importance of collecting data on sleep duration on weekends, to capture both a negative and a positive social jetlag. Moreover, our findings highlighted the need to characterize potential factors influencing social jetlag, to gain an understanding of the characteristics of the individuals who exhibit a long positive social jetlag. In particular, work patterns; mental illness; and psychosocial aspects such as stress, fatigue, and mood, as potential factors underlying social jetlag warrant investigation.

Although not the primary objective of our analyses, differences in sleep duration according to BMI were noted. In particular, individuals with a lower BMI were more likely to be in the highest tertile of sleep duration. These findings were in line with the observations made previously, in a cross-sectional analysis of NHANES 2007–2008 [27]. They were also consistent with an earlier analysis of the NDNS RP [9], wherein Potter and colleagues found that for every additional hour of sleep, there was a 0.46 kg/m^2^ and 0.9 cm reduction in BMI and waist circumference, respectively. However, in our study, we observed that despite the inverse association between sleep duration and BMI in the univariate models, long sleep duration on weekdays was associated with a lower healthy dietary pattern score, compared to normal sleep duration.

To date, inconsistencies remain about the best categorization of sleep duration. These discrepancies have been highlighted by a recent review by Al-Khatib and colleagues [4]. The current National Sleep Foundation defines normal sleep for adults as a range of 7–9 h, while sleep duration equal or below 6 h is deemed to be short sleep, and 10 h and above is defined as long sleep [39]. Other studies utilize 9 h as the cut-off, for defining long sleep duration [1]. In the present study, we assessed model fit based on multiple approaches including categorizing sleep duration into tertiles and use of a quadratic term. We found that the model that included tertiles of sleep duration on weekdays vs. weekends provided the best fit when examining the association between sleep duration and diet. This was despite the unequal sample distribution within the tertiles, which arose as a result of the Stata xtile command being confronted with ties [40]. Redefining the lowest and highest tertile using 6 h and 9 h, respectively, as cut-offs did not improve the model fit or change the value of the coefficient. In contrast, when analyzing social jetlag data, categorizing the social jetlag variable into tertiles did not provide the best model fit. Moreover, the categorization was difficult to interpret, as it was unclear as to what constitutes a normal range of social jetlag. Consequently, the final model with the quadratic term was selected, as it provided a better fit and interpretation. The implication of using the different methodologies to define sleep duration or social jetlag warrant further investigation. Such research may be important in understanding potential differences in the definition of short, normal, and long sleep duration or social jetlag in different global regions.

### 4.2. Strengths and Weaknesses of the Study

A main strength of the current study is the national representativeness of the survey sample, and the use of detailed dietary data, which was collected over 3–4 days. Data on sleep duration on weekends were also available, which allowed us to adjust for sleep duration on weekends and explore how sleep compensation on weekends may confound or modify the association between sleep during weekdays, and overall diet. Previously such analysis had not been possible, as most nutritional surveys did not collect data on sleep during weekends [1]. This is an important strength, as research has shown that the relationship between sleep duration and diet may differ when considering sleep duration on weekends [27]. A further strength of the present study was the use of multiple imputation to account for missing data, compared to previous studies, which focused only on individuals with complete data [9]. Multiple imputation has the advantage of providing a narrower confidence interval, and reducing bias associated with the use of a selective sample [41].

Concerning limitations; one of the main limitations of NDNS RP is the cross-sectional design, which does not permit investigating the direction of causality in the association between sleep and dietary patterns. Indeed, whilst sleep duration has been shown to influence eating behavior and nutritional status in a number of experimental studies, certain foods or nutrients may equally impact sleep duration and quality, as reviewed by Dashti [7] and Pot [42]. In addition, the NDNS RP included data from all participants, regardless as to whether the dietary data collected during the three to four days of food recordings, was reflective of habitual intake [43]. This aspect of the study design meant that differentiating between under-reporters and under-consumers (for instance due to ill health), may not be possible [43]. Nevertheless, mis-reporting is a common limitation of dietary surveys and cohorts. A further limitation was the absence of data on physical activity, shift work, timing of sleep onset, or sleep quality in the adult population. Finally, in the NDNS RP, data on sleep duration were self-reported, and no objective measures on sleep duration and quality were available in the adult population. Hence, findings from our study may differ from studies that utilize more objective measures of sleep duration [27].

## 5. Conclusions and Future Directions

In conclusion, long sleep duration on weekdays and a positive social jetlag are associated, inversely, with a healthy dietary pattern. Future studies should investigate the factors influencing differences in dietary patterns, based on different sleep duration categories. Moreover, research should address how sleep duration relates to timing of food intake and explore differences in eating occasion-related dietary patterns. Such research will permit the development of more effective dietary strategies to improve the overall diet of individuals, at different points of the sleep duration spectrum.

## Figures and Tables

**Table 1:** Study population characteristics of the complete case study sample by tertiles of sleep duration on weekdays. Continuous data is shown as mean and standard errors. Categorical data is shown as counts and percentages.

Factor	Level	Sleep Duration Tertile			*p*-Value *
		T1 (Short ≤ 7 h)	T2 (Normal > 7 and ≤ 8 h)	T3 (Long > 8 h)	
N unweighted ^†^		1350	798	285	
Sleep duration on weekdays (hours)		6.3 (0.9)	7.8 (0.2)	9.2 (0.7)	<0.001
Sleep duration on weekends (hours)		6.9 (1.5)	8.1 (1.1)	9.0 (1.3)	<0.001
Social jetlag (hours)		0.6 (1.2)	0.2 (1.1)	−0.2 (1.2)	<0.001
Sleep duration on weekends	T1 (short)	911 (67.5%)	87 (10.9%)	27 (9.5%)	<0.001
	T2 (normal)	261 (19.3%)	490 (61.4%)	33 (11.6%)	
	T3 (long)	178 (13.2%)	221 (27.7%)	225 (78.9%)	
Healthy dietary pattern		0.0 (1.6)	0.1 (1.7)	−0.4 (1.5)	0.060
Sugar, milk & bread dietary pattern		0.1 (1.4)	−0.1 (1.3)	0.0 (1.3)	0.055
Snacks dietary pattern		0.0 (1.4)	0.0 (1.3)	−0.1 (1.3)	0.102
Total energy (MJ) diet only		7.7 (2.5)	7.6 (2.4)	7.1 (2.3)	0.011
Age (years)		43.9 (11.9)	41.5 (12.4)	38.7 (13.1)	<0.001
BMI (kg/m^2^)		28.2 (5.5)	27.2 (5.4)	26.6 (5.3)	<0.001
Sex	Male	610 (45%)	322 (40%)	87 (30%)	0.017
	Female	740 (55%)	476 (60%)	198 (70%)	
Ethnic group	White	1262 (94%)	731 (92%)	267 (94%)	0.742
	Non-white	88 (6%)	67 (8%)	18 (6%)	
Cigarette Smoking Status	Current cigarette smoker	378 (28%)	186 (23%)	89 (31%)	0.307
	Ex-regular cigarette smoker	274 (20%)	163 (21%)	51 (18%)	
	Never regular cigarette smoker	698 (52%)	449 (56%)	145 (51%)	
Socioeconomic Status	Managerial & professional occupations	564 (42%)	343 (43%)	92 (32%)	0.094
	Intermediate occupations	127 (9%)	76 (10%)	25 (9%)	
	Small employers & own account workers	128 (9%)	88 (11%)	38 (13%)	
	Lower supervisory & technical occupations	148 (11%)	52 (6%)	26 (9%)	
	Semi-routine & routine occupations	360 (27%)	218 (27%)	98 (35%)	
	Never worked & long-term unemployed	23 (2%)	21 (3%)	6 (2%)	
Mental Illness	No	432 (93%)	195 (91%)	93 (88%)	0.159
	Yes	31 (7%)	19 (9%)	13 (12%)	

* *p*-Values are provided taking into consideration complex survey design and weighted sample. Singleton units were centred to enable estimation of standard errors based on the distance of strata from the grand mean. ^†^ N unweighted for complete-case model including mental illness as a covariate is 783. Abbreviations: T, tertile; N, number; MJ, millijoules; BMI, body mass index; kg, kilograms; m, meters.

**Table 2:** Proportion of consumers within each food group within each sleep duration tertile.

Food Group	Sleep Duration on Weekdays Tertile			*p*-Value *
	T1	T2	T3	
	*n* (%)	*n* (%)	*n* (%)	
Bacon & ham	832 (61.6%)	483 (60.5%)	175 (61.4%)	0.610
Beef, veal, &, dishes	692 (51.3%)	399 (50%)	141 (49.5%)	0.823
Beer, lager, cider, perry	445 (33%)	238 (29.8%)	63 (22.1%)	0.003
Biscuits	858 (63.6%)	504 (63.2%)	169 (59.3%)	0.276
Brown granary & wheatgerm bread	481 (35.6%)	263 (33%)	80 (28.1%)	0.020
Buns, cakes, pastries, fruit, pies	698 (51.7%)	388 (48.6%)	125 (43.9%)	0.260
Burgers & kebabs	175 (13%)	111 (13.9%)	40 (14%)	0.546
Butter	454 (33.6%)	268 (33.6%)	104 (36.5%)	0.865
Cheese	822 (60.9%)	500 (62.7%)	163 (57.2%)	0.897
Chicken & turkey dishes	884 (65.5%)	548 (68.7%)	198 (69.5%)	0.695
Chips, fried, roast, potatoes, &, potato	858 (63.6%)	497 (62.3%)	192 (67.4%)	0.140
Chocolate confectionery	605 (44.8%)	365 (45.7%)	122 (42.8%)	0.300
Coated chicken	221 (16.4%)	125 (15.7%)	48 (16.8%)	0.587
Crisps	659 (48.8%)	404 (50.6%)	129 (45.3%)	0.386
Dry weight beverages	181 (13.4%)	96 (12%)	28 (9.8%)	0.026
Eggs & egg dishes	681 (50.4%)	395 (49.5%)	158 (55.4%)	0.185
Fruit	1021 (75.6%)	633 (79.3%)	196 (68.8%)	0.040
Fruit juice	483 (35.8%)	314 (39.3%)	94 (33%)	0.222
High fibre breakfast cereals	584 (43.3%)	376 (47.1%)	114 (40%)	0.492
Ice-cream	247 (18.3%)	145 (18.2%)	43 (15.1%)	0.656
Lamb & dishes	168 (12.4%)	113 (14.2%)	37 (13%)	0.422
Liver dishes	61 (4.5%)	33 (4.1%)	9 (3.2%)	0.834
Low fat spread	239 (17.7%)	142 (17.8%)	49 (17.2%)	0.233
Low fat spread not PUFA ^†^	75 (5.6%)	42 (5.3%)	11 (3.9%)	0.903
Meat pies & pastries	316 (23.4%)	183 (22.9%)	71 (24.9%)	0.735
Nuts & seeds	241 (17.9%)	153 (19.2%)	38 (13.3%)	0.019
Oily fish	279 (20.7%)	188 (23.6%)	40 (14%)	0.018
One percent milk	28 (2.1%)	14 (1.8%)	9 (3.2%)	0.125
Other bread	127 (9.4%)	88 (11%)	24 (8.4%)	0.898
Other breakfast cereals	389 (28.8%)	262 (32.8%)	97 (34%)	0.629
Other margarine fats & oils	110 (8.1%)	93 (11.7%)	25 (8.8%)	0.103
Other meat & meat products	189 (14%)	108 (13.5%)	41 (14.4%)	0.635
Other potatoes, potato, salads, dishes	252 (18.7%)	144 (18%)	45 (15.8%)	0.547
Other milk & cream	910 (67.4%)	543 (68%)	195 (68.4%)	0.942
Other white, fish, shellfish, fish, dishes	442 (32.7%)	292 (36.6%)	96 (33.7%)	0.457
Pasta, rice & other cereals	1021 (75.6%)	617 (77.3%)	216 (75.8%)	0.303
Pork & dishes	259 (19.2%)	151 (18.9%)	61 (21.4%)	0.201
Puddings	301 (22.3%)	170 (21.3%)	51 (17.9%)	0.091
PUFA margarine oils	34 (2.5%)	26 (3.3%)	6 (2.1%)	0.635
Reduced fat spread not PUFA	606 (44.9%)	361 (45.2%)	131 (46%)	0.671
Reduced fat spread PUFA	190 (14.1%)	103 (12.9%)	37 (13%)	0.643
Salad & other raw vegetables	957 (70.9%)	595 (74.6%)	199 (69.8%)	0.376
Sausages	469 (34.7%)	254 (31.8%)	103 (36.1%)	0.900
Savoury sauces, pickles & condiments	1076 (79.7%)	656 (82.2%)	229 (80.4%)	0.629
Semi skimmed milk	970 (71.9%)	604 (75.7%)	196 (68.8%)	0.435
Skimmed milk	212 (15.7%)	107 (13.4%)	36 (12.6%)	0.623
Soft drinks low calorie	489 (36.2%)	319 (40%)	113 (39.6%)	0.650
Soft drinks not low calorie	659 (48.8%)	396 (49.6%)	150 (52.6%)	0.657
Soup homemade & retail	421 (31.2%)	269 (33.7%)	112 (39.3%)	0.064
Spirits & liqueurs	201 (14.9%)	105 (13.2%)	27 (9.5%)	0.063
Sugar confectionery	196 (14.5%)	123 (15.4%)	33 (11.6%)	0.813
Sugars, preserves & sweet spreads	839 (62.1%)	512 (64.2%)	169 (59.3%)	0.313
Tea, coffee & water	1325 (98.1%)	786 (98.5%)	280 (98.2%)	0.529
Vegetables not raw	1200 (88.9%)	719 (90.1%)	248 (87%)	0.226
White bread	1069 (79.2%)	632 (79.2%)	230 (80.7%)	0.328
White fish, coated or fried	276 (20.4%)	177 (22.2%)	71 (24.9%)	0.027
Wholemeal bread	478 (35.4%)	301 (37.7%)	97 (34%)	0.094
Whole milk	268 (19.9%)	144 (18%)	76 (26.7%)	0.329
Wine	431 (31.9%)	274 (34.3%)	80 (28.1%)	0.161
Yogurt, fromage frais & dairy dessert	504 (37.3%)	321 (40.2%)	98 (34.4%)	0.354

* *p*-Values were obtained from chi-square test for categorical variables and are provided taking into consideration complex survey design and weighted sample. ^†^ PUFA, Polyunsaturated fatty acids. Abbreviations: T, tertile; *n*, number.

**Table 3:** Coefficient estimates from the multiple regression models of the association between sleep duration on weekdays and healthy dietary pattern as the outcome.

Model	Parameter		Coefficient	Lower CI	Upper CI	*p*-Value *
Crude	Sleep Weekday	T1	−0.07	−0.33	0.19	0.595
*R*^2^ = 0.012	Sleep Weekday	T2 (Reference)	0.00			
	Sleep Weekday	T3	−0.60	−0.95	−0.26	0.001
	Sleep Weekend	T1	−0.17	−0.42	0.09	0.207
	Sleep Weekend	T2 (Reference)	0.00			
	Sleep Weekend	T3	−0.09	−0.38	0.21	0.553
	Intercept		0.29	0.10	0.49	0.004
Model 1 ^†^	Sleep Weekday	T1	−0.12	−0.36	0.12	0.325
*R*^2^ = 0.18	Sleep Weekday	T2 (Reference)	0.00			
	Sleep Weekday	T3	−0.48	−0.82	−0.15	0.005
	Sleep Weekend	T1	−0.10	−0.33	0.13	0.395
	Sleep Weekend	T2 (Reference)	0.00			
	Sleep Weekend	T3	0.10	−0.18	0.38	0.473
	Sex	women vs. men	0.63	0.46	0.80	<0.001
	Ethnicity	non-white vs. white	0.40	0.15	0.64	0.002
	Age	(years)	0.04	0.04	0.05	<0.001
	Total Energy Intake	(MJ)	0.19	0.15	0.24	<0.001
	Intercept		−3.42	−3.93	−2.91	<0.001
Model 2 ^‡^	Sleep Weekday	T1	−0.12	−0.35	0.11	0.301
*R*^2^ = 0.28	Sleep Weekday	T2 (Reference)	0.00			
	Sleep Weekday	T3	−0.45	−0.78	−0.12	0.007
	Sleep Weekend	T1	−0.04	−0.26	0.18	0.729
	Sleep Weekend	T2 (Reference)	0.00			
	Sleep Weekend	T3	0.17	−0.10	0.44	0.215
	Sex	women vs. men	0.57	0.40	0.73	<0.001
	Ethnicity	non-white vs. white	0.34	0.10	0.59	0.006
	Smoking status	Ex-regular cigarette smoker	0.71	0.47	0.95	<0.001
		Never smoker	0.81	0.61	1.01	<0.001
	Socioeconomic Status	Q1	0.00			
		Q2	−0.34	−0.60	−0.08	0.010
		Q3	−0.38	−0.65	−0.10	0.007
		Q4	−0.24	−0.52	0.04	0.093
		Q5	−0.64	−0.84	−0.44	<0.001
		Q6	0.17	−0.42	0.75	0.579
	Age	(years)	0.04	0.04	0.05	<0.001
	BMI	(kg/m^2^)	−0.02	−0.04	−0.01	0.001
	Total Energy Intake	(MJ)	0.17	0.13	0.22	<0.001
	Intercept		−2.94	−3.59	−2.28	<0.001
Complete-cases model ^§^	Sleep Weekday	T1	−0.12	−0.35	0.11	0.304
	Sleep Weekday	T2 (Reference)				
*R*^2^ = 0.29	Sleep Weekday	T3	−0.41	−0.77	−0.06	0.021
	Sleep Weekend	T1	−0.04	−0.26	0.18	0.742
	Sleep Weekend	T2 (Reference)				
	Sleep Weekend	T3	0.18	−0.10	0.45	0.199
	Sex	women vs. men	0.52	0.35	0.69	0
	Ethnicity	non-white vs. white	0.33	0.13	0.54	0.001
	Smoking status	Ex-regular cigarette smoker	0.68	0.42	0.94	<0.001
		Never smoker	0.77	0.55	0.99	<0.001
	Socioeconomic Status (NS-SEC)	Q1				
		Q2	−0.34	−0.62	−0.07	0.014
		Q3	−0.39	−0.67	−0.11	0.007
		Q4	−0.21	−0.52	0.09	0.175
		Q5	−0.69	−0.90	−0.49	<0.001
		Q6	0.09	−0.54	0.71	0.785
	Age (years)		0.04	0.04	0.05	<0.001
	BMI	(kg/m^2^)	−0.02	−0.04	−0.01	<0.001
	Total Energy Intake	(MJ)	0.18	0.13	0.22	<0.001
	Intercept		−2.91	−3.58	−2.24	<0.001

Note: * *p*-Values were obtained taking into consideration complex survey design and weighted sample. Variables with *p* ≤ 0.01 were deemed to be significant. ^†^ Model 1 adjusted for sex, ethnicity, National Statistics Socio-economic Classification (NS-SEC), age, smoking status, and mean daily energy intake. Sample size consisted of an unweighted *n* = 2697. ^‡^ Model 2 additionally adjusted for sex, ethnicity, NS-SEC, age, smoking status, mean daily energy intake, and BMI. § Sample size consisted of an unweighted *n* = 2433. Abbreviations: T, tertile; Q, quantile; BMI, Body mass index; kg, kilograms; m, meters; MJ, millijoules.

**Table 4:** Coefficient estimates from the multiple regression models of the association between social jetlag and healthy dietary pattern as the outcome.

Model	Parameter		Coefficient	Lower CI	Upper CI	*p*-Value *
Crude Model	Social jetlag		0.12	0.04	0.20	0.002
*R*^2^ = 0.01	Social jetlag squared		−0.04	−0.06	−0.01	0.002
	Intercept		0.11	0.01	0.21	0.036
Model 1 ^†^	Social jetlag		0.12	0.04	0.19	0.003
*R*^2^ = 0.19	Social jetlag squared		−0.04	−0.05	−0.02	<0.001
	Sex	women vs. men	0.64	0.46	0.81	<0.001
	Ethnicity	non-white vs. white	0.37	0.12	0.63	0.004
	Age	(years)	0.04	0.03	0.05	<0.001
	Total Energy Intake	(MJ)	0.21	0.16	0.25	<0.001
	Constant		−3.59	−4.07	−3.11	<0.001
Model 2 ^‡^	Social jetlag		0.11	0.03	0.18	0.005
*R*^2^ = 0.29	Social jetlag squared		−0.03	−0.04	−0.01	0.005
	Sex	women vs. men	0.57	0.41	0.73	<0.001
	Ethnicity	non-white vs. white	0.32	0.08	0.57	0.011
	Smoking status	Ex-regular cigarette smoker	0.73	0.48	0.97	<0.001
		Never smoker	0.81	0.60	1.01	<0.001
	Socioeconomic Status	Q1	0.00			
	(NS-SEC)	Q2	−0.35	−0.61	−0.08	0.011
		Q3	−0.37	−0.64	−0.11	0.006
		Q4	−0.28	−0.55	0.00	0.050
		Q5	−0.61	−0.81	−0.42	<0.001
		Q6	0.17	−0.42	0.77	0.564
	Age	(years)	0.04	0.03	0.05	<0.001
	BMI	(kg/m^2^)	−0.02	−0.04	−0.01	0.001
	Total Energy Intake	(MJ)	0.18	0.14	0.22	<0.001
	Intercept		−3.05	−3.67	−2.43	<0.001
Complete-cases ^§^	Social jetlag		0.11	0.03	0.19	0.005
*R*^2^ = 0.29	Social jetlag squared		−0.03	−0.05	−0.01	0.002
	Sex	women vs. men	0.52	0.35	0.69	<0.001
	Ethnicity	non-white vs. white	0.31	0.10	0.52	0.004
	Smoking status	Ex-regular cigarette smoker	0.70	0.44	0.95	<0.001
		Never smoker	0.76	0.55	0.98	<0.001
	Socioeconomic Status	Q1				
		Q2	−0.34	−0.63	−0.06	0.017
		Q3	−0.39	−0.66	−0.11	0.006
		Q4	−0.25	−0.55	0.05	0.103
		Q5	−0.65	−0.86	−0.45	<0.001
		Q6	0.09	−0.55	0.72	0.787
	Age	(years)	0.04	0.03	0.05	<0.001
	BMI	(kg/m^2^)	−0.02	−0.04	−0.01	<0.001
	Total Energy Intake	(MJ)	0.19	0.14	0.23	<0.001
	Intercept		−3.01	−3.66	−2.36	<0.001

Note: * *p*-Values were obtained taking into consideration complex survey design and weighted sample. Variables with *p* ≤ 0.01 were deemed to be significant. ^†^ Model 1 adjusted for sex, ethnicity, Socio-economic Classification (NS-SEC), age, smoking status, and mean daily energy intake. Sample size consisted of an unweighted *n* = 2697. ^‡^ Model 2 additionally adjusted for sex, ethnicity, NS-SEC, age, smoking status, mean daily energy intake, and BMI. ^§^ Sample size consisted of an unweighted *n* = 2433. Abbreviations: MJ, millijoules; BMI, Body Mass Index; kg, kilograms; m, meters; Q, quantile.

## Supplementary Materials

The following are available online at http://www.mdpi.com/2072-6643/10/9/1131/s1. Table S1: Coefficient estimates from the regression model of the association between sleep duration on weekdays or sleep lag and the healthy dietary pattern including mental illness as a covariate. Variables with *p* ≤ 0.01 were deemed to be significant, Table S2: Coefficient estimates from the regression model of the association between sleep duration on weekdays and the healthy dietary pattern excluding sleep on weekends as a covariate. Variables with *p* ≤ 0.01 were deemed to be significant, Table S3: Coefficient estimates from the regression model of the association between sleep duration on weekdays and the healthy dietary pattern. Lower tertiles defined using ≤6 h as cut-off and upper tertile defined using ≥9 h as cut-off. Variables with *p* ≤ 0.01 were deemed to be significant.
