# Supplementary material for: Long Sleep Duration and Social Jetlag Are Associated Inversely with a Healthy Dietary Pattern in Adults: Results from the UK National Diet and Nutrition Survey Rolling Programme Y1–4
Source: Nutrients. 2018 Aug 21;10(9):1131. doi: 10.3390/nu10091131 (PMC6163907; doi:10.3390/nu10091131)
Supplement: Supplementary file 1 [file nutrients-10-01131-s001.pdf]

Supplementary Material

Table 1S Coefficient estimates from the regression model of the association between sleep duration on weekdays (Model 3a) or social jetlag (Model 3b) and the healthy dietary pattern, including mental illness as a covariate. Variables with P ≤0.01 were deemed to be significant. Singleton Units re-assigned to nearest relevant stratum.

| Model 3 | | | Coefficient* | Lower | Upper | p-value† |
|---|---|---|---|---|---|---|
| A | Sleep Weekday | T1 | -0.12 | -0.35 | 0.11 | 0.292 |
| | Sleep Weekday | T2 (Reference) | 0.00 | . | . | . |
| | Sleep Weekday | T3 | -0.44 | -0.77 | -0.11 | 0.009 |
| | Sleep Weekend | T1 | -0.04 | -0.26 | 0.18 | 0.728 |
| | Sleep Weekend | T2 (Reference) | 0.00 | . | . | . |
| | Sleep Weekend | T3 | 0.17 | -0.10 | 0.44 | 0.217 |
| | Sex | women vs men | 0.57 | 0.41 | 0.73 | <0.001 |
| | Ethnicity | non-white vs white | 0.35 | 0.10 | 0.59 | 0.006 |
| | Smoking status | Ex-regular cigarette smoker | 0.70 | 0.45 | 0.94 | <0.001 |
| | | Never smoker | 0.79 | 0.59 | 1.00 | <0.001 |
| | Socioeconomic Status | Q1 | 0.00 | . | . | . |
| | | Q2 | -0.34 | -0.60 | -0.08 | 0.010 |
| | | Q3 | -0.35 | -0.63 | -0.07 | 0.014 |
| | | Q4 | -0.24 | -0.52 | 0.04 | 0.091 |
| | | Q5 | -0.62 | -0.82 | -0.42 | <0.001 |
| | | Q6 | 0.15 | -0.44 | 0.74 | 0.613 |
| | Age (years) | | 0.04 | 0.03 | 0.05 | <0.001 |
| | BMI (kg/m2) | | -0.02 | -0.04 | -0.01 | 0.001 |
| | Total Energy Intake (MJ) | | 0.17 | 0.13 | 0.21 | <0.001 |
| | Mental Illness | Yes | -0.24 | -0.71 | 0.22 | 0.305 |
| | Intercept | | -2.85 | -3.52 | -2.17 | <0.001 |
| B | Social jetlag | | 0.11 | 0.03 | 0.18 | 0.007 |
| | Social jetlag squared | | -0.03 | -0.04 | -0.01 | 0.007 |
| | Sex | women vs men | 0.57 | 0.41 | 0.73 | <0.001 |
| | Ethnicity | non-white vs white | 0.33 | 0.08 | 0.57 | 0.010 |
| | Smoking status | Ex-regular cigarette smoker | 0.72 | 0.48 | 0.96 | <0.001 |
| | | Never smoker | 0.80 | 0.59 | 1.00 | <0.001 |
| | Mental Illness | Yes | -0.11 | -0.59 | 0.36 | 0.634 |
| | Socioeconomic Status | Q1 | 0.00 | . | . | . |
| | | Q2 | -0.35 | -0.61 | -0.08 | 0.010 |
| | | Q3 | -0.36 | -0.63 | -0.09 | 0.009 |
| | | Q4 | -0.28 | -0.56 | 0.00 | 0.049 |
| | | Q5 | -0.61 | -0.81 | -0.40 | <0.001 |
| | | Q6 | 0.17 | -0.43 | 0.77 | 0.574 |
| | Age | (years) | 0.04 | 0.03 | 0.05 | <0.001 |
| | BMI | (kg/m2) | -0.02 | -0.04 | -0.01 | 0.001 |
| | Total Energy Intake | (MJ) | 0.18 | 0.14 | 0.22 | <0.001 |
| | Intercept | | -3.00 | -3.65 | -2.36 | <0.001 |

*Note:*
* Model 3 adjusted for sex, ethnicity, NS-SEC, age, smoking status, mean daily energy intake, BMI, and mental illness. Sample size consisted of an unweighted $n$=2697. † P-values were obtained taking into consideration complex survey design and weighted sample. Variables with $P \leq 0.01$ were deemed to be significant.
Abbreviations: T, tertile; Q, quantile; BMI, Body mass index; kg, kilograms; m, meters; MJ millijoules

Table 2S Coefficient estimates from the regression model of the association between sleep duration on weekdays and the healthy dietary pattern, excluding sleep at weekends as a covariate. Variables with P ≤ 0.01 were deemed to be significant. Singleton Units re-assigned to nearest relevant stratum.

| | | Coefficient* | Lower | Upper | p-value† |
|---|---|---|---|---|---|
| Sleep Weekday | T1 | -0.16 | -0.36 | 0.03 | 0.102 |
| Sleep Weekday | T2 (Reference) | 0.00 | . | . | . |
| Sleep Weekday | T3 | -0.37 | -0.66 | -0.07 | 0.015 |
| Sex | women vs men | 0.56 | 0.40 | 0.73 | 0.000 |
| Ethnicity | non-white vs white | 0.35 | 0.09 | 0.60 | 0.007 |
| Smoking status | Ex-regular cigarette smoker | 0.71 | 0.46 | 0.95 | 0.000 |
| | Never smoker | 0.81 | 0.60 | 1.01 | 0.000 |
| Socioeconomic Status | Q1 | 0.00 | . | . | . |
| | Q2 | -0.35 | -0.62 | -0.09 | 0.010 |
| | Q3 | -0.37 | -0.65 | -0.10 | 0.007 |
| | Q4 | -0.24 | -0.52 | 0.04 | 0.094 |
| | Q5 | -0.64 | -0.84 | -0.44 | 0.000 |
| | Q6 | 0.17 | -0.41 | 0.75 | 0.568 |
| Age (years) | | 0.04 | 0.04 | 0.05 | <0.001 |
| BMI (kg/m$^2$) | | -0.02 | -0.04 | -0.01 | 0.001 |
| Total Energy Intake (MJ) | | 0.17 | 0.13 | 0.22 | <0.001 |
| Intercept | | -2.86 | -3.54 | -2.18 | <0.001 |

*Note:*

* Model adjusted for sex, ethnicity, NS-SEC, age, smoking status, mean daily energy intake, BMI, and mental illness. Sample size consisted of an unweighted *n*=2697. † P-values were obtained taking into consideration complex survey design and weighted sample. Variables with $P \leq 0.01$ were deemed to be significant.
Abbreviations: T, tertile; Q, quantile; BMI, Body mass index; kg, kilograms; m, meters; MJ millijoules

Table 3S Coefficient estimates from the regression model of the association between sleep duration on weekdays and the healthy dietary pattern. Lower tertiles defined using ≤ 6h as cut-off and upper tertile defined using ≥ 9h as cut-off. Variables with P ≤ 0.01 were deemed to be significant. Singleton Units re-assigned to nearest relevant stratum.

| | | Coefficient* | Lower | Upper | p-value† |
|---|---|---|---|---|---|
| Sleep Weekday | T1 | -0.09 | -0.34 | 0.17 | 0.503 |
| Sleep Weekday | T2 (Reference) | 0.00 | . | . | . |
| Sleep Weekday | T3 | -0.47 | -0.82 | -0.11 | 0.010 |
| Sleep Weekend | T1 | -0.06 | -0.32 | 0.20 | 0.665 |
| Sleep Weekend | T2 (Reference) | 0.00 | . | . | . |
| Sleep Weekend | T3 | 0.23 | -0.04 | 0.51 | 0.100 |
| Sex | women vs men | 0.57 | 0.41 | 0.74 | <0.001 |
| Ethnicity | non-white vs white | 0.34 | 0.09 | 0.58 | 0.008 |
| Smoking status | Ex-regular cigarette smoker | 0.71 | 0.47 | 0.95 | <0.001 |
| | Never smoker | 0.80 | 0.60 | 1.01 | <0.001 |
| Socioeconomic Status | Q1 | 0.00 | . | . | . |
| | Q2 | -0.35 | -0.61 | -0.09 | 0.009 |
| | Q3 | -0.37 | -0.64 | -0.10 | 0.007 |
| | Q4 | -0.24 | -0.52 | 0.03 | 0.085 |
| | Q5 | -0.63 | -0.83 | -0.44 | <0.001 |
| | Q6 | 0.17 | -0.44 | 0.77 | 0.589 |
| Age (years) | | 0.04 | 0.04 | 0.05 | <0.001 |
| BMI (kg/m2) | | -0.02 | -0.04 | -0.01 | 0.001 |
| Total Energy Intake (MJ) | | 0.17 | 0.13 | 0.21 | <0.001 |
| Intercept | | -3.01 | -3.65 | -2.38 | <0.001 |

*Note:*
* Model adjusted for sleep duration on weekends, sex, ethnicity, NS-SEC, age, smoking status, mean daily energy intake, and BMI. Sample size consisted of an unweighted *n*=2697. † P-values were obtained taking into consideration complex survey design and weighted sample. Variables with *P* ≤ 0.01 were deemed to be significant. Abbreviations: T, tertile; Q, quantile; BMI, Body mass index; kg, kilograms; m, meters; MJ millijoules
